# The impact of maternal immune activation on embryonic brain development

**DOI:** 10.3389/fnins.2023.1146710

**Published:** 2023-03-06

**Authors:** Francesca McEwan, Jocelyn D. Glazier, Reinmar Hager

**Affiliations:** Division of Evolution, Infection and Genomic Sciences, School of Biological Sciences, Manchester Academic Health Science Centre, Faculty of Biology, Medicine and Health, University of Manchester, Manchester, United Kingdom

**Keywords:** maternal immune activation, neurogenesis, neurodevelopment, proliferation, schizophrenia, autism spectrum disorder, cortical lamination

## Abstract

The adult brain is a complex structure with distinct functional sub-regions, which are generated from an initial pool of neural epithelial cells within the embryo. This transition requires a number of highly coordinated processes, including neurogenesis, i.e., the generation of neurons, and neuronal migration. These take place during a critical period of development, during which the brain is particularly susceptible to environmental insults. Neurogenesis defects have been associated with the pathogenesis of neurodevelopmental disorders (NDDs), such as autism spectrum disorder and schizophrenia. However, these disorders have highly complex multifactorial etiologies, and hence the underlying mechanisms leading to aberrant neurogenesis continue to be the focus of a significant research effort and have yet to be established. Evidence from epidemiological studies suggests that exposure to maternal infection *in utero* is a critical risk factor for NDDs. To establish the biological mechanisms linking maternal immune activation (MIA) and altered neurodevelopment, animal models have been developed that allow experimental manipulation and investigation of different developmental stages of brain development following exposure to MIA. Here, we review the changes to embryonic brain development focusing on neurogenesis, neuronal migration and cortical lamination, following MIA. Across published studies, we found evidence for an acute proliferation defect in the embryonic MIA brain, which, in most cases, is linked to an acceleration in neurogenesis, demonstrated by an increased proportion of neurogenic to proliferative divisions. This is accompanied by disrupted cortical lamination, particularly in the density of deep layer neurons, which may be a consequence of the premature neurogenic shift. Although many aspects of the underlying pathways remain unclear, an altered epigenome and mitochondrial dysfunction are likely mechanisms underpinning disrupted neurogenesis in the MIA model. Further research is necessary to delineate the causative pathways responsible for the variation in neurogenesis phenotype following MIA, which are likely due to differences in timing of MIA induction as well as sex-dependent variation. This will help to better understand the underlying pathogenesis of NDDs, and establish therapeutic targets.

## 1. Introduction

Neuronal development is a highly orchestrated process in which the proliferation, differentiation and migration of neuronal cells allow distinct functional sub-regions to form, which eventually comprise the complex structure and function of the adult brain (Urbán and Guillemot, 2014; Mira and Morante, 2020). Compelling data suggests aberrant neurogenesis is a fundamental convergence point in the etiology of all neurodevelopmental disorders (NDDs), such as schizophrenia and autism spectrum disorder (ASD; Ernst, 2016; Fan and Pang, 2017). This is evidenced following genetic studies, which show that a high proportion of genes linked to NDDs are implicated in cellular proliferation and differentiation (Ernst, 2016).

Neurodevelopmental disorders have complex multifactorial origins; believed to be triggered by a combination of genetic and environmental factors (De Felice et al., 2015; Wilson et al., 2022). A rapidly growing and dominant hypothesis in this field is exposure to immune activation during early development *in utero* (Kinney et al., 2010; Feigenson et al., 2014). This was first evidenced following naturally occurring epidemics, such as the 1957 influenza epidemic in Finland, where an increased proportion of the population, who were in their second trimester of gestational development at the time of the epidemic, were later diagnosed with schizophrenia (Mednick et al., 1988; Sham et al., 1992).

Animal models have been developed to establish the mechanisms underlying the link between exposure to maternal infection and increased risk of developing NDDs (Woods et al., 2021; Bao et al., 2022). Several models involve direct administration of microorganisms, such as influenza, to the pregnant rodent (Garbett et al., 2012; Jacobsen et al., 2021). However, following the understanding that it is maternal immune activation (MIA) rather than the pathogen itself that increases the risk for NDDs (Patterson, 2002; Shi et al., 2005; Brown, 2006; Estes and McAllister, 2016), MIA is most commonly induced by bacterial or viral mimetics, lipopolysaccharide (LPS) or polyinosinic:polycytidylic acid [poly(I:C)], respectively (Meyer, 2014; Bergdolt and Dunaevsky, 2019; Woods et al., 2021; Bao et al., 2022). This stimulates the release of pro-inflammatory cytokines in the maternal plasma through activation of toll-like receptors and elicits adolescent and adult behavioral deficits reminiscent to NDD symptoms in the offspring (Meyer, 2014; Estes and McAllister, 2016; Bergdolt and Dunaevsky, 2019).

Maternal immune activation is commonly induced during mid to late gestation, which is defined as a critical period of brain development, during which neurogenesis can be influenced by adverse environmental conditions (Selemon and Zecevic, 2015; Dehorter and Del Pino, 2020). Therefore, it is reasonable to hypothesize that defects in neurogenesis are at the root of MIA-induced brain and behavioral deficits, which has been the focus of recent studies (Ben-Reuven and Reiner, 2021; Canales et al., 2021; Long et al., 2021; Tsukada et al., 2021; Loayza et al., 2022; Yu et al., 2022). However, results in outcome data often vary between MIA models, and hence the direct actions of MIA on embryonic neurogenesis and neuronal migration remain often largely unclear. This review summarizes the changes to the proliferation and differentiation of neurons, neuronal migration and cortical lamination in the embryonic rodent brain following MIA induction, and develops hypotheses about the link between these aspects of neurogenesis and aberrant brain phenotypes.

## 2. Modeling maternal immune activation

Animal models of human disorders should have face validity, meaning the model has similar endophenotypes to the human disease, construct validity, related to the biological deficit causing the disease, and predictive validity, defined as the similarity between model and patient in response to treatment (Crawley, 2012; Silverman et al., 2022). Strong evidence for predictive and face validity has been reported in the MIA model [reviewed extensively by Bergdolt and Dunaevsky (2019) and Haddad et al. (2020)], which is demonstrated by behavioral outputs recapitulating NDD-like symptoms in the offspring. For example, animals display altered amphetamine sensitivity when assessed through locomotor activity (Zager et al., 2012; Gray et al., 2019; Weber-Stadlbauer et al., 2021), as well as cognitive dysfunction, demonstrated by visual recognition and spatial memory deficits (Savanthrapadian et al., 2013; MacDowell et al., 2017; Richetto et al., 2017a; Lorusso et al., 2022). These are translatable to the positive and cognitive symptoms of schizophrenia, including psychotic agitation and dysfunctional working memory, respectively (Redrobe et al., 2010; van den Buuse, 2010; Batinić et al., 2016). Despite this, the biological deficits associated with MIA have been suggested to reflect specific aspects of NDDs, rather than recapitulating the biological phenotypes as a whole (Haddad et al., 2020). For example, MIA offspring brains exhibit shifts in excitatory versus inhibitory signaling systems, including defects in parvalbumin-containing γ-aminobutyric acid (GABA)ergic neurons (Zhang and van Praag, 2015; Canetta et al., 2016; Vojtechova et al., 2021), as well as alterations in the glutamatergic N-methyl-D-aspartate receptor subunit composition (Rahman et al., 2017; Hao et al., 2019). Deficits in glutamatergic and GABAergic neurotransmission are present in the brains of patients with schizophrenia and ASD (Gonzalez-Burgos et al., 2015; Balu, 2016; Rahman et al., 2020; Schoonover et al., 2020; Strube et al., 2020) and are believed to underlie the cognitive deficits associated with NDDs (Bojesen et al., 2021; Kumar et al., 2021). In comparison, few studies report changes to dopaminergic gene expression (Woods et al., 2021), which, until recently, dominated the field of schizophrenia research and remains the primary target of antipsychotic treatment (Coyle et al., 2010; Stahl, 2018; McCutcheon et al., 2020). However, current treatment lacks efficacy in ameliorating the negative and cognitive symptoms of schizophrenia (Fusar-Poli et al., 2015; McCutcheon et al., 2020), and hence, the MIA model may provide a useful tool in helping to identify therapeutic targets for these symptoms.

## 3. Typical neurogenesis

**FIGURE 1:**
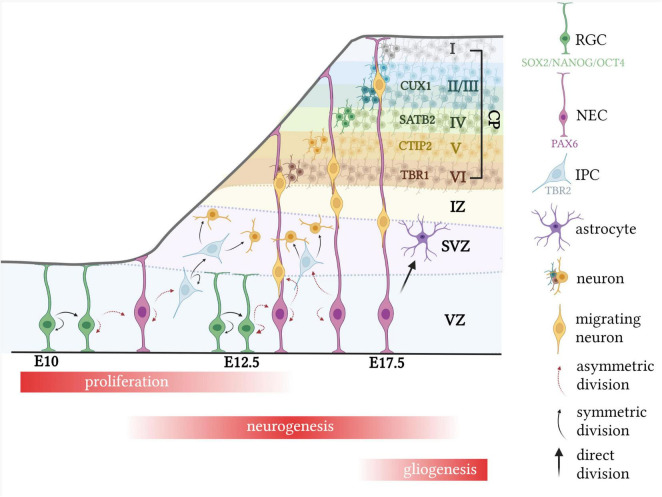
Typical murine corticogenesis. Following expansion of the NEC pool, division of NECs begin to switch from symmetric to asymmetric, forming one RGC and one NEC. RGCs then divide either asymmetrically to generate two RGCs, or symmetrically to form one RGC and an IPC or neuron. IPCs divide only symmetrically to form a pair of IPCs or neurons. Migrating neurons use radial glial cells as scaffolds, to migrate up to the cortical plate, which expands in an “inside-out” manner, with the deep layer IV neurons forming first, at around E12.5. During late corticogenesis, RGCs begin to directly divide into glial cells, such as astrocytes. CP, cortical plate; CTIP2, COUP TF1-interacting protein 2; CUX1, cut like homeobox 1; E, embryonic day; SATB2, special AT-rich sequence-binding protein 2; SOX2, SRY-box 2; PAX6, paired box protein Pax-6; TBR1, T-box brain transcription factor; TBR2, T-box brain protein 2; IPC, intermediate progenitor cell; NANOG, nanog homeobox; NEC, neural epithelial cell. Created with BioRender.com.

Neurodevelopment begins with the formation of the neural tube, where an initial pool of neuroepithelial cells (NECs) divide symmetrically until a sufficient number have formed (Figure 1; Stiles and Jernigan, 2010; Egger et al., 2011; Semple et al., 2013). At around embryonic day (E) 10–12 in the mouse telencephalon, division of NECs begins to switch from symmetric to asymmetric, forming one NEC and a radial glial cell (RGC; Dennis et al., 2016). The gradual switch from proliferation to differentiation is associated with cell cycle changes including decreased re-entry and increased cell cycle exit, as well as parameter alterations, such as lengthening of the cell cycle, or G1 phase specifically (Sommer and Rao, 2002; Ohnuma and Harris, 2003; Lancaster and Knoblich, 2012; Hardwick et al., 2015; Szűcs et al., 2020).

This transition is highly regulated by the expression of specific transcription factors, which may be used to trace the lineage of NEC to mature neuron (Urbán and Guillemot, 2014; Stevanovic et al., 2021). It is widely known that SRY-box 2 (SOX2), octamer-binding transcription factor 4 (OCT4) and homeobox protein NANOG (NANOG) are involved in maintaining pluripotency among stem cells (Figure 1; Ahmed et al., 2009; Desai and Pethe, 2020). The transition from NEC to RGC is associated with a decrease in expression of these pluripotent transcription factors and a concomitant increase in paired box protein pax-6 (PAX6) expression, which has received a lot of attention due to its essential role in controlling neurogenesis (Sansom et al., 2009; Suter et al., 2009; Zhang et al., 2010; Manuel et al., 2015). Despite the glial-like properties of RGCs, including certain molecular and cytological features, they can divide asymmetrically to form one RGC and either a neuron or an intermediate progenitor cell (IPC), identified as direct and indirect neurogenesis, respectively, and are responsible for the formation of all cortical neurons as well as several glial cell lineages (Figure 1; Beattie and Hippenmeyer, 2017). IPCs, also known as basal progenitors, have been uniquely associated with the subventricular zone (SVZ), which is located above the ventricular zone (VZ), and, unlike NECs and RGCs, divide only symmetrically a limited number of times (1–3) to produce neurons (Figure 1; Kowalczyk et al., 2009; Mira and Morante, 2020). For this reason, it has been suggested that IPCs function to increase the number of neurons and size of the SVZ, which becomes one of the two limited neurogenic regions in the adult (Smart et al., 2002; Götz and Huttner, 2005; Mira and Morante, 2020). RGCs, which eventually give rise to IPCs, transiently express neurogenin 2, which is the transcriptional target of T-box brain protein 2 (TBR2) and is expressed in IPCs. Hence, differentiation of RGCs to IPCs to neurons is associated with decreased PAX6 and increased TBR2 cellular density, followed by increased expression of post-mitotic neuron markers, such as neuron-specific class III beta-tubulin (TUJ1) or T-box brain transcription factor 1 (TBR1; Figure 1; Sun and Hevner, 2014; Manuel et al., 2015; Guo et al., 2021; Kim et al., 2021).

## 4. MIA-induced defects

Although embryonic neurogenesis and neuronal migration are highly controlled processes, they are defined as a critical period of neurodevelopment, which can be influenced by adverse environmental challenges (Fan and Pang, 2017). Accordingly, current evidence suggests MIA affects the proliferation of NECs, the differentiation of those cells into neurons and the migration of neurons to form distinct regions (De Miranda et al., 2010; Carpentier et al., 2011, 2013; Stolp et al., 2011; Gumusoglu et al., 2017; Ben-Reuven and Reiner, 2021).

### 4.1. Neurogenesis

Several studies show decreased proliferation in the fetal cortex acutely following MIA, demonstrated by reduced phosphohistone H3 (PHH3), a mitotically active cell marker shortly after (2–8 h) LPS administration (Table 1; Carpentier et al., 2011; Stolp et al., 2011; Kim et al., 2017; Braun et al., 2019). This is supported by nucleotide uptake studies, where the administration of a thymidine analog allows the synthesis of DNA, and hence, the proportion of cell divisions, to be tracked. For example, a reduction in single nucleotide uptake was reported when administered at 2–22 h post-LPS administration, which provides evidence for reduced proliferation in the MIA embryonic cortex (Cui et al., 2009; Stolp et al., 2011; Carpentier et al., 2013). Double-labeled thymidine studies enable a more precise determination of proliferation kinetics, where the time interval between the two nucleotides defines the cellular readout (Solius et al., 2021). Studies report decreased double-labeled cells within the first 24 h following both LPS and poly(I:C)-induced MIA, when an interval of over 10 h was used between nucleotide administration, indicating that there is a reduction in the number of cells which have re-entered the cell cycle (De Miranda et al., 2010; Carpentier et al., 2013). This is supported by findings of an increased quit fraction in these cells, which represents the proportion of cells that have left the cell cycle (Table 1; Carpentier et al., 2013). However, when an interval of 2.5 h was used, there was no significant change in the proportion of double labeled cells within the first 8 h after poly(I:C) injection and this was increased after 24 h (Ben-Reuven and Reiner, 2021). In contrast to 10 h, 2.5 h may be long enough to allow cells to exit S-phase, but not to re-enter, and hence, double-labeled cells in this instance are more likely to represent cells that have stayed in S-phase, rather than re-entered, suggesting that the length of S-phase has changed (Ben-Reuven and Reiner, 2021). Nucleotide dilution assays can also indicate proliferation state, where the nucleotide becomes diluted through cell divisions, and hence, high threshold cells represent cells that have not divided but are still in S-phase, and a low threshold signal is indicative of cells that have undergone multiple cell divisions. Studies report significantly decreased number of high threshold cells acutely following MIA in the fetal cortex (De Miranda et al., 2010; Ben-Reuven and Reiner, 2021), and De Miranda et al. (2010) showed that this was concordant with no change in the number of low threshold cells, which would be expected to increase following increased cell cycle divisions. They hence proposed that there is increased cell cycle exit, lending support to the theory of decreased proliferation acutely following MIA (De Miranda et al., 2010; Carpentier et al., 2011, 2013; Stolp et al., 2011; Ben-Reuven and Reiner, 2021).

**TABLE 1:** Cell cycle phenotypes in fetal MIA offspring brains relative to vehicle controls.

Cellular phenotype	Marker	Location	Change	Time after MIA brain taken or nucleotide administration (T)	Induction (route, commercial source), rodent, embryonic day	Sex	References
Active cell cycle	Ki67+	Cortex (VZ)	↓	5 days	30 mg/kg PIC (i.p., Sigma) C57BL/6N mice, 12.5	M, F	Canales et al., 2021
		CGE	↓	48 h	150 μg/kg LPS (i.p., Sigma), GAD65-GFP C57BL/6 mice, 15.5 + 16.5	M, F	Lacaille et al., 2019
Active mitosis	PHH3+	Cortex (VZ)	↓	5 days	30 mg/kg PIC (i.p., Sigma) C57BL/6N mice, 12.5	M, F	Canales et al., 2021
		Cortical neurosphere (*in vitro*)	↑	7 days	10 mg/kg PIC (i.p., Sigma), Sprague Dawley rats, 8.5	M, F	Baines et al., 2020
		Cortex	↓	2 h	60 μg/kg (LPS, Sigma), C57BL/6N mice	ND	Carpentier et al., 2011
		Cortex	NS	6 h	20 mg/kg PIC (i.p., Sigma), C57BL/6 mice, 12.5	ND	Ben-Reuven and Reiner, 2021
		Cortex	↑	24 h			
		Cortex	M: ↓ F: NS	2 h	60 μg/kg LPS (i.p., Sigma), C57BL/6 mice, 12.5	M, F	Braun et al., 2019
		Cortex (VZ)	↓	8 h	10 μg/kg LPS (i.p., Sigma), C57BL/6 mice, 13.5	ND	Stolp et al., 2011
		Cortex (SVZ)	NS				
		Cortex (VZ)	NS	48 h			
		Cortex (SVZ)	NS				
Cells in S-phase	Thymidine analog+	Cortex	↓	2 h (T^1^), 24 h	60 μg/kg LPS (i.p., Sigma), C57BL/6 mice, 12.5	ND	Carpentier et al., 2013
			↓	22 h (T^2^), 24 h			
			NS	0 h (T^1^), PD0	5 mg/kg PIC (i.p., Sigma), C57BL/6 mice, 16	ND	De Miranda et al., 2010
			↑	7.5 h (T^2^), 8 h	20 mg/kg PIC (i.p., Sigma), C57BL/6 mice, 12.5	ND	Ben-Reuven and Reiner, 2021
		Cortex	NS	24 h + 36 + 72 h (T^1^), 4 days	2.5 or 25 μg/kg LPS (i.p., Sigma), Sprague Dawley rats, 14	ND	Chao et al., 2016
		Cortical neurosphere (*in vitro*)	↑	7 days, 14 days (T^1^)	10 mg/kg PIC (i.p., Sigma), Sprague Dawley rats, 8.5	M, F	Baines et al., 2020
		Cortex (VZ)	↓	24 h (T^1^), 8 h	10 μg/kg LPS (i.p., Sigma), C57BL/6 mice, 13.5	ND	Stolp et al., 2011
		Cortex	↑	24 h (T^1^), 2 days	5 μg recombinant mouse IL-6 (i.p., R&D), CD1 mice, 13.5	ND	Gallagher et al., 2013
		Cortex (VZ + SVZ)	↑	24 h (T^1^), 4 days			
		Cortex (IZ)	NS				
		Cortex (CP)	↓				
		CGE	↓	0 h (T^1^), 48 h	150 μg/kg LPS (i.p., Sigma), GAD65-GFP C57BL/6 mice, 15.5 + 16.5	M, F	Lacaille et al., 2019
Re-entered or stayed in S-phase	Thymidine analog+/thymidine analog+	Cortex	↓	2 h (T^1^), 22 h (T^2^), 26 h	60 μg/kg LPS (i.p., Sigma), C57BL/6 mice, 12.5	ND	Carpentier et al., 2013
			↓	0 h (T^1^) 10 h (T^2^), PD0	5 mg/kg PIC (i.p., Sigma), C57BL/6 mice, 16	ND	De Miranda et al., 2010
			NS	5 h (T^1^), 7.5 h (T^2^), 8 h	20 mg/kg PIC (i.p., Sigma), C57BL/6 mice, 12.5	ND	Ben-Reuven and Reiner, 2021
			↑	24 h (T^1^), 26.5 h (T^2^), 27 h	20 mg/kg PIC (i.p., Sigma), C57BL/6 mice, 12.5		
Exited S-phase	Thymidine analog+/thymidine analog−	Cortex	↑	5 h (T^1^), 7.5 h (T^2^), 8 h	20 mg/kg PIC (i.p., Sigma), C57BL/6 mice, 12.5	ND	Ben-Reuven and Reiner, 2021
			NS	24 h (T^1^), 26.5 h (T^2^), 27 h	20 mg/kg PIC (i.p., Sigma), C57BL/6 mice, 12.5		
Quit fraction	Ki67-/thymidine analog+	Cortex	↑	2 h (T^1^), 24 h	60 μg/kg LPS (i.p., Sigma), C57BL/6 mice, 12.5	ND	Carpentier et al., 2013
Proportion of cell divisions	Thymidine analog dilution assay	Cortex	↓ T^HIGH^	6 h (T^1^), 24 h	20 mg/kg PIC (i.p., Sigma), C57BL/6 mice, 12.5	ND	Ben-Reuven and Reiner, 2021
			↓ T^HIGH^ NS T^LOW^	24 h (T^1^), 3 days	5 mg/kg PIC (i.p., Sigma), C57BL/6 mice, 15 + 16 + 17	ND	De Miranda et al., 2010

CGE, caudal ganglionic eminence; CP, cortical plate; F, female; IZ, intermediate zone; h, hours; LPS, lipopolysaccharide; M, male; mg/ml, milligram per milliliter; mg/kg, milligram per kilogram dam weight; ND, not described; PD, postnatal day; PHH3, phosphohistone H3; PIC, poly(I:C); SVZ, subventricular zone; T, time after MIA of nucleotide administration; μg/kg, microgram per kilogram dam weight; VZ, ventricular zone; ↑, significantly increased; ↓, significantly decreased; +, positive stained cell; −, negative stained cell.

**FIGURE 2:**
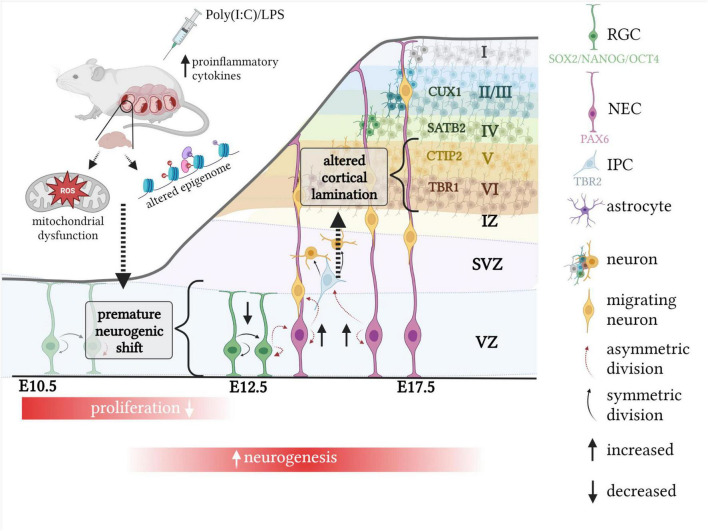
The impact of MIA on murine corticogenesis. Exposure to MIA whilst *in utero* causes an acute neurogenesis defect, associated with a decreased proportion of symmetric, proliferative divisions and increased number of asymmetric, neurogenic divisions. This results in cortical lamination defects which are dependent on the timing of MIA exposure. When induced at E12.5, increased neuron production at the expense of proliferation is associated with altered density of earlier born cortical layers, such as VI and V, which are defined by the transcription factors TBR1 and CTIP2, respectively. Mitochondrial dysfunction as well as an altered epigenome may explain the acute neurogenesis defect following MIA. Created with BioRender.com.

The defective proliferation phenotype appears to be associated with a premature acceleration in neurogenesis, where cells are exhibiting a higher proportion of neurogenic divisions at the expense of self-renewal (Figure 2). This is demonstrated by cell cycle parameter changes recorded acutely following MIA, including a shortening of S-phase, which has been associated with a commitment to neuron production (Salomoni and Calegari, 2010; Arai et al., 2011; Mi et al., 2018; Ben-Reuven and Reiner, 2021). In accordance, it appears more NECs are dividing asymmetrically to form RGCs, IPCs or neurons, which is demonstrated by increased expansion of the RGC (PAX6) population (De Miranda et al., 2010; Tsukada et al., 2021) and elevated proportion of newly formed IPCs (Tsukada et al., 2021), contributing to indirect neurogenesis. Furthermore, studies report increased number of RGCs giving rise to a post-mitotic neuron and RGC, otherwise known as direct neurogenesis (Ben-Reuven and Reiner, 2021). Although the route of neurogenesis appears to differ between studies, where there is a discrepancy in the proportion of newly formed IPCs (Table 2; De Miranda et al., 2010; Ben-Reuven and Reiner, 2021; Tsukada et al., 2021), it is clear that MIA acutely promotes neural differentiation within the fetal brain (Figure 2).

**TABLE 2:** Cell fate phenotypes in fetal MIA offspring cortex relative to controls.

Cellular phenotype	Marker	Change	Time after MIA brain taken or nucleotide administration (T)	Induction (route, commercial source), rodent, embryonic day	Sex	References
NECs	SOX2+	↓	5 days	30 mg/kg PIC (i.p., Sigma) C57BL/6N mice, 12.5	M, F	Canales et al., 2021
		↑	24 h	20 mg/kg PIC (i.p., Sigma), C57BL/6 mice, 12.5	ND	Ben-Reuven and Reiner, 2021
		↑	6 days			
NECs in S-phase	SOX2+/thymidine analog+	↑	24 h (T^1^), 4 days	5 μg recombinant mouse IL-6 (i.p., R&D), CD1 mice, 13.5	ND	Gallagher et al., 2013
RGCs	PAX6+	↑	24 h	20 mg/kg PIC (i.p. Sigma) C57BL/6J mice, 12.5	ND	Tsukada et al., 2021
		NS	48 h			
		↑ in SVZ	6 days			
		↑	24 h (T^1^), 3 days	5 mg/kg PIC (i.p., Sigma), C57BL/6 mice, 15 + 16 + 17	ND	De Miranda et al., 2010
		NS	8 h	20 mg/kg PIC (i.p., Sigma), C57BL/6 mice, 12.5	ND	Ben-Reuven and Reiner, 2021
		NS	24 h			
		↓	5 days	30 mg/kg PIC (i.p., Sigma) C57BL/6N mice, 12.5	M, F	Canales et al., 2021
		↓	4 days	100 μg/kg (i.p., Sigma), Sprague Dawley rat, 15–16	ND	Cunningham et al., 2013
RGC in S-phase	PAX6+/thymidine analog+	↑	6 h (T^1^), 24 h	20 mg/kg PIC (i.p., Sigma), C57BL/6 mice, 12.5	ND	Ben-Reuven and Reiner, 2021
		NS	4 days (T^1^), 5 days	20 mg/kg PIC (i.p., Sigma), ddY mice, 9.5	ND	Soumiya et al., 2011
		NS	4 days (T^1^), 5.5 days			
		NS	4 days (T^1^), 6 days			
		↓	6 days (T^1^), 5 days			
		↓	6 days (T^1^), 5.5 days			
		NS	6 days (T^1^), 6 days			
RGC neurogenic divisions	PAX6+→ PAX6+/TUJ1+	↑	8 h	20 mg/kg PIC (i.p., Sigma), C57BL/6 mice, 12.5	ND	Ben-Reuven and Reiner, 2021
		NS	24 h			
Newly formed IPCs	PAX6+/TBR2+	↑	24 h	20 mg/kg PIC (i.p. Sigma) C57BL/6J mice, 12.5	ND	Tsukada et al., 2021
		NS	48 h			
		↓	24 h (T^1^), 3 days	5 mg/kg PIC (i.p., Sigma), C57BL/6 mice, 15 + 16 + 17	ND	De Miranda et al., 2010
		NS	24 h	20 mg/kg PIC (i.p., Sigma), C57BL/6 mice, 12.5	ND	Ben-Reuven and Reiner, 2021
IPCs	TBR2+	↑ SVZ	6 days	20 mg/kg PIC (i.p. Sigma) C57BL/6J mice, 12.5	ND	Tsukada et al., 2021
		NS	24 h	20 mg/kg PIC (i.p., Sigma), C57BL/6 mice, 12.5	ND	Ben-Reuven and Reiner, 2021
		↓	4 days	75 μg/kg LPS (i.p., Sigma), C57BL/6 mice, 14.5	ND	Wu et al., 2018
		↓	4 days	100 μg/kg (i.p., Sigma), rat (strain ND), 15 + 16	ND	Cunningham et al., 2013
IPCs in S-phase	TBR2+/thymidine analog+	NS	6 h (T^1^), 24 h	20 mg/kg PIC (i.p., Sigma), C57BL/6 mice, 12.5	ND	Ben-Reuven and Reiner, 2021
		NS	4 days (T^1^), 5 days	20 mg/kg PIC (i.p., Sigma), ddY mice, 9.5	ND	Soumiya et al., 2011
		NS	4 days (T^1^), 5.5 days			
		NS	4 days (T^1^), 6 days			
		NS	6 days (T^1^), 5 days			
		NS	6 days (T^1^), 5.5 days			
		NS	6 days (T^1^), 6 days			

F, female; h, hours; IPC, immediate progenitor cell; LPS, lipopolysaccharide; M, male; mg/kg, milligram per kilogram dam weight; ND, not described; NEC, neural epithelial cell; PAX6, paired box protein Pax-6; PHH3, phosphohistone H3; PIC, poly(I:C); RGC, radial glial cell; SOX2, SRY-box 2; SVZ, subventricular zone; T, time after MIA of nucleotide administration; TBR2, T-box brain protein 2; TUJ1, beta-III tubulin; VZ, ventricular zone; ↑, significantly increased; ↓, significantly decreased; +, positive stained cell; −, negative stained cell.

Further evidence of a neurogenesis defect has been demonstrated at a delayed time point following MIA, including a reduction in proliferation after 5–6 days (Soumiya et al., 2011; Canales et al., 2021), which is in contrast to reports of increased proliferation in cortical neurospheres taken from offspring brains one week following MIA (Table 1; Baines et al., 2020). However, the use of an *in vitro* approach in the latter experiment may not directly reflect *in vivo* phenotypes, and hence, it is not known whether the differences in proliferation between these two studies is due to gestational timing differences or experimental approach. In comparison to the acute time point, dysregulated proliferation 3–5 days following MIA is predominantly associated with decreased proportion of RGCs and IPCs (Table 2; Soumiya et al., 2011; Cunningham et al., 2013; Canales et al., 2021).

It is likely that discrepancies in methodology between studies, including experimental approach, i.e., *in vivo* or *in vitro*, species and strain of model, source and dosage of immunostimulant and timing of MIA induction, contribute to differential outcomes (Kowash et al., 2019; Bao et al., 2022). Timing of MIA induction may be particularly important in this case due to the rapid and precise nature of embryonic neurogenesis (Urbán and Guillemot, 2014). The majority of studies referenced in this review expose pregnant mice to MIA at E12.5–13.5 (Maekawa et al., 2005; Osumi et al., 2008; Carpentier et al., 2011; Stolp et al., 2011; Gallagher et al., 2013; Choi et al., 2016; Braun et al., 2019; Ben-Reuven and Reiner, 2021; Canales et al., 2021; Tsukada et al., 2021), which, in typical murine neurodevelopment, coincides with significant changes in PAX6 density (Englund et al., 2005; Duan et al., 2013; Ben-Reuven and Reiner, 2021). It is thus not surprising that MIA at this time point primarily affects the PAX6 cell population. Several studies reported an acute increase in the proportion of PAX6 cells, which appears to reverse three days after MIA (De Miranda et al., 2010; Soumiya et al., 2011; Ben-Reuven and Reiner, 2021; Canales et al., 2021; Tsukada et al., 2021). In contrast, MIA at an early gestational time point, such as E8.5, exhibits a pro-proliferation phenotype (Baines et al., 2020). Differences in brain and behavioral phenotype depending on timing of gestational MIA exposure have been consistently reported and may translate to the development of distinct NDDs in humans, hence highlighting the requirement for further research (Meyer et al., 2006; Guma et al., 2022; Nakamura et al., 2022).

An increasing body of evidence suggests that there are sex-dependent effects in developing resilience or susceptibility to neurodevelopmental insults, reported in both patients and animal models (Palmer et al., 2017; Nugent et al., 2018; Hodes and Epperson, 2019; May et al., 2019; Vojtechova et al., 2021; Woodward and Coutellier, 2021). It is therefore essential for sex to be treated as an independent variable within MIA models (Coiro and Pollak, 2019). However, a number of studies referenced in this section did not indicate which sex was used for offspring experiments, nor how sex was determined (Tables 1–3), and it may therefore be assumed that in these instances, sex was disregarded, with male and female being treated as one group. Data shows a disparity in sex response to MIA in a number of developmental stages and systems (Haida et al., 2019), from the adult behavioral phenotype (Gogos et al., 2020; Zhao et al., 2021) to the acute inflammatory response (Barke et al., 2019). We may thus expect sex differences in neurogenesis. This view is further supported by evidence of decreased active mitosis in male but not female MIA cortices (Braun et al., 2019), as well as results showing that male neural progenitors have an increased tendency to differentiate *in vitro* (Baines et al., 2020). Hence, the pooling of both sexes into one group may often mask a sex-dependent effect, which in turn may lead to variation in proliferation phenotypes across studies.

**TABLE 3:** Cortical lamination changes in MIA fetal offspring brains in contrast to vehicle controls.

Layer	Cell marker	Location	Direction of change in MIA	Time after MIA brain taken or nucleotide administration (T)	Induction (commercial source), rodent, strain, embryonic day	Sex	References
VI	TBR1+	Cortex	NS	48 h	20 mg/kg PIC (i.p. Sigma), C57BL/6J mice, 12.5	ND	Tsukada et al., 2021
		Cortex	NS	6 days	20 mg/kg PIC (i.p., Sigma), C57BL/6 mice, 12.5	ND	Ben-Reuven and Reiner, 2021
		Cortex	NS	24 h	20 mg/kg PIC (i.p., Sigma), C57BL/6 mice, 13.5		
		Cortex	↓	5 days	30 mg/kg PIC (i.p., Sigma), C57BL/6N mice, 12.5	M, F	Canales et al., 2021
		Cortical plate	NS	4 days	75 μg/kg LPS (i.p., Sigma), C57BL/6, 14.5	ND	Wu et al., 2018
		Cortex	2.5 μg/kg: NS 25 μg/kg: ↓	4 days	2.5 or 25 μg/kg LPS (i.p., Sigma), Sprague Dawley rats, 14	M, F	Chao et al., 2016
		Cortex (blocks 0–5)	↑	6 days	20 mg/kg PIC (i.p., Sigma) C57BL/6 mice, 12.5	M	Choi et al., 2016
		Cortex (blocks 6–7)	↓				
		Cortex (blocks 8–10)	NS				
	TBR1+/thymidine analog+	Cortex	NS	6 h (T^1^), 6 days	20 mg/kg PIC (i.p., Sigma), C57BL/6 mice, 12.5	ND	Ben-Reuven and Reiner, 2021
	TBR1 (thickness)	Cortex	NS	5 days	30 mg/kg PIC (i.p., Sigma), C57BL/6N mice, 12.5	M, F	Canales et al., 2021
V	CTIP2+	Cortex	↓	5 days	30 mg/kg PIC (i.p., Sigma), C57BL/6N mice, 12.5	M, F	Canales et al., 2021
		Cortex	2.5 μg/kg: NS 25 μg/kg: ↓	4 days	2.5 or 25 μg/kg LPS (i.p., Sigma), Sprague Dawley rats, 14	M, F	Chao et al., 2016
		Cortex	↑	6 days	20 mg/kg PIC (i.p., Sigma), C57BL/6 mice, 12.5	ND	Ben-Reuven and Reiner, 2021
	CTIP2+/thymidine analog	Cortex	↑	6 h (T^1^), 24 h	20 mg/kg PIC (i.p., Sigma), C57BL/6 mice, 13.5	ND	Ben-Reuven and Reiner, 2021
			↑	6 h (T^1^), 6 days	20 mg/kg PIC (i.p., Sigma), C57BL/6 mice, 12.5		
	CTIP2 (thickness)	Cortex	↓	5 days	30 mg/kg PIC (i.p., Sigma), C57BL/6N mice, 12.5	M, F	Canales et al., 2021
IV	SATB2+	Cortex	NS	5 days	30 mg/kg PIC (i.p., Sigma), C57BL/6N mice, 12.5	M, F	Canales et al., 2021
		Cortical plate	↓	4 days	75 μg/kg LPS (i.p., Sigma), C57BL/6, 14.5	ND	Wu et al., 2018
		Cortex	↓	6 days	20 mg/kg PIC (i.p., Sigma), C57BL/6 mice, 12.5	ND	Ben-Reuven and Reiner, 2021
		Cortex (block 2)	↑	6 days	20 mg/kg PIC (i.p., Sigma), C57BL/6 mice, 12.5	M	Choi et al., 2016
		Cortex (blocks 3–4)	↓				
		Cortex (blocks 0–1, 5–10)	NS				
	SATB2+/thymidine analog+	Cortex	↓	24 h (T^1^), 4 days	5 μg recombinant mouse IL-6 (i.p., R&D), CD1 mice	ND	Gallagher et al., 2013
		Cortex	↑	6 h (T^1^), 6 days	20 mg/kg PIC (i.p., Sigma), C57BL/6 mice, 12.5	ND	Ben-Reuven and Reiner, 2021
	SATB2 (thickness)	Cortex	↑	5 days	30 mg/kg PIC (i.p., Sigma), C57BL/6N mice, 12.5	M, F	Canales et al., 2021
II–III	CUX1+	Cortex	NS	6 days	20 mg/kg PIC (i.p., Sigma), C57BL/6 mice, 12.5	ND	Ben-Reuven and Reiner, 2021
	CUX1+/thymidine analog+	Cortex	NS	6 h (T^1^), 6 days	20 mg/kg PIC (i.p., Sigma), C57BL/6 mice, 12.5	ND	Ben-Reuven and Reiner, 2021
	CUX1 (thickness)	Cortex	NS	5 days	30 mg/kg PIC (i.p., Sigma), C57BL/6N mice, 12.5	M, F	Canales et al., 2021

CTIP2, COUP TF1-interacting protein 2; CUX1, cut like homeobox 1; F, female; h, hours; LPS, lipopolysaccharide; M, male; mg/kg, milligram per kilogram dam weight; ND, not described; PIC, poly(I:C); SATB2, special AT-rich sequence-binding protein 2; T, time after MIA of nucleotide administration; TBR1, T-box brain transcription factor 1; ↑, significantly increased; ↓, significantly decreased; +, positive stained cell; −, negative stained cell.

### 4.2. Neuronal migration

Neuronal migration is a complex process, which involves the coordination of neuronal branching and extension with cellular movement, and is guided by a number of vital signaling molecules and stimuli (Khodosevich and Monyer, 2011; Cooper, 2013; Buchsbaum and Cappello, 2019). Neurons migrate *via* two distinct mechanisms, radial and tangential, which are predominantly utilized by cortical projection neurons and GABAergic interneurons, respectively. Radial migration describes the process used by neurons migrating from the VZ, where radial glial “guides” are used as a scaffold for migration (Figure 1; Molyneaux et al., 2007; Stiles and Jernigan, 2010; Perez-Garcia and O’Leary, 2016). On the other hand, tangential migration involves neurons migrating from five main proliferative regions, in a manner parallel to the plial surface and perpendicular to radially migrating neurons, which thereby increases neuronal diversity in the brain (Marín, 2015).

Evidence suggests MIA offspring have neuronal migratory defects (Shi et al., 2009; Soumiya et al., 2011; Gumusoglu et al., 2017), such as a transient delay in cellular migration at E13.5–E14.5 in cortices exposed to MIA on E9.5 (Soumiya et al., 2011). GABAergic progenitors also show defective migration at E14 following administration of IL-6 to the pregnant dam (Gumusoglu et al., 2017), which may be linked to dysregulated expression of molecules required for interneuron migration, such as *Nkx2.1, Nrp1, Trkb*, and *Arx* as well as the *Dlx* family of genes (Oskvig et al., 2012; Nakamura et al., 2019, 2022).

Aside from these studies, there has been minimal research regarding neuronal migration in the MIA-offspring fetal brain, presumably due to the difficultly in researching cellular migration *in vivo*. However, neuronal migration in the murine neocortex takes place during mid-to-late gestation (Ayala et al., 2007), which often coincides with the timing of MIA insult. Further, genes involved in neuronal migration were differentially expressed in MIA prenatal non-candidate gene sets (Oskvig et al., 2012; Lombardo et al., 2018). Future research on the affected migratory processes following MIA is therefore necessary to elucidate NDD pathogenesis.

### 4.3. Cortical lamination

The proliferation and migration of neurons is instrumental in defining complex structures within the brain, one of which, the neocortex, is characterized by the lamination of neurons into six distinct layers (Kostović and Judaš, 2007; Guarnieri et al., 2019; Wu et al., 2021). During early murine cortical development (around E10.5), the first wave of neuronal migration from the VZ and SVZ forms the thin, preplate layer which later splits into the superficial marginal zone and the inner subplate, allowing the cortical plate to form in between (Figure 1). The cortical plate is then expanded in a tightly orchestrated, “inside-out” manner to form the characteristic six-layered structure of the cortex (Figure 1). These layers are defined by distinct cellular morphologies and densities, with each projecting to designated regions of the brain (Harris and Shepherd, 2015; Lodato and Arlotta, 2015). In brief, during early murine corticogenesis (E12.5), a wave of migrating neurons forms the corticothalamic layer, otherwise known as layer VI, which can be defined by the expression of TBR1. Layers V to II then migrate successively, in temporal waves, past earlier-born neurons, forming the subcerebral layer (V), the pyramidal layer (IV), and upper callosal layers (III-II). These layers are recognized by the expression of the transcription factors B-cell lymphoma/leukemia 11B (CTIP2), special AT-rich sequence-binding protein (SATB2), and cut like homeobox 1 (CUX1), respectively and are formed on E13.5–E14.5 (Figure 1; Molyneaux et al., 2007; Ben-Reuven and Reiner, 2021).

The densities of these layers are often altered following MIA exposure, particularly the earlier born, deep-layer neurons. However, there are differences in the direction of density change (Table 3; Gallagher et al., 2013; Chao et al., 2016; Choi et al., 2016; Wu et al., 2018; Ben-Reuven and Reiner, 2021; Canales et al., 2021), most likely due to minor discrepancies in timing of MIA induction as well as the gestational time point of lamination. The latter theory is supported by a study which administered the thymidine analog, BrdU, daily on E13.75–E16.75 after poly(I:C) on E9.5 and tracked layer density according to cell birth date. Although the deep-layer born neurons were consistently increased between E14.75 and E16.75 in MIA offspring brains, this was not evident at E13.75, and the group which had the most densely populated upper-layer fluctuated between poly(I:C) and control animals depending on embryonic day (Soumiya et al., 2011). This suggests poly(I:C) may affect the timing at which layer-specific neurons are generated (Choi et al., 2016), where there appears to be increased production of deep-layer neurons, later in corticogenesis, when this would be expected to be diminished. A number of studies support the notion of an immature phenotype in poly(I:C) animals during late gestational development, including evidence of overlapped TBR1 and CTIP2 neurons at E20, which are clearly distinguishable in vehicle controls at this time point (Chao et al., 2016), as well as localization of PAX6 and TBR2 cells in the SVZ during late corticogenesis, which would typically only be localized in the VZ at this stage (Tsukada et al., 2021).

Changes to lamination could be explained by the aforementioned acute and premature shift from symmetric to asymmetric divisions in the MIA fetal brain, where more neurons are exiting the cell cycle and are maturing to becoming CTIP2/TBR1 positive (Figure 2). These cells are typically formed at E12.5/E13.5 which coincides with timing of MIA induction of the majority of studies referenced in this review (Maekawa et al., 2005; Osumi et al., 2008; Carpentier et al., 2011; Stolp et al., 2011; Gallagher et al., 2013; Braun et al., 2019; Ben-Reuven and Reiner, 2021; Canales et al., 2021; Tsukada et al., 2021).

## 5. Causative mechanisms

### 5.1. Paired box protein pax-6

Overall, the PAX6 positive RGCs are the most affected population of cells within the embryonic brain following immune insult (Table 2), and a number of studies additionally report a change in PAX6 protein expression (De Miranda et al., 2010; Wu et al., 2018; Ben-Reuven and Reiner, 2021; Canales et al., 2021). PAX6 is a highly dose dependent transcription factor, where increased expression favors increased neurogenic divisions, yet knockout studies have also shown that it is essential for progenitor proliferation (Maekawa et al., 2005; Osumi et al., 2008; Sansom et al., 2009; Mi et al., 2018). Hence, the acute increase in PAX6 protein expression or relative intensity, which has been reported in MIA offspring (De Miranda et al., 2010; Ben-Reuven and Reiner, 2021), may be directly linked to the premature switch to neurogenic phenotype. In contrast, studies report decreased PAX6 protein expression 4–5 days following immune insult (Wu et al., 2018; Canales et al., 2021). This could be the result of a compensatory mechanism due to early depletion of the progenitor pool, therefore reversing the effect of MIA, which would support the aforementioned immature lamination at this time point.

Research indicates that PAX6, and other transcriptional regulators of neurogenesis, may be directly controlled by inflammatory mediators which are upregulated in the MIA offspring brain (Loayza et al., 2022), such as microglia (Cunningham et al., 2013) and cytokines (Walter et al., 2011; Borsini et al., 2015). However, a number of studies report no changes to inflammation status within the fetal MIA offspring brain (see Hameete et al., 2021), yet neurogenesis is defective across the prenatal timeline (Tables 1–3). Epigenetic mechanisms are believed to mediate the prolonged effects of MIA on the offspring brain and behavioral phenotypes (Bergdolt and Dunaevsky, 2019), and hence, considering epigenetics is critical for healthy neurogenesis (Albert et al., 2017; Albert and Huttner, 2018), could provide a basis for putative causative mechanisms.

### 5.2. Epigenetics

Epigenetic modifications, comprising DNA methylation, histone modifications and non-coding RNAs, enable a change in transcriptional response without editing the underlying DNA (Goldberg et al., 2007; Capell and Berger, 2013). As well as playing vital roles in cellular differentiation and other “typical” developmental mechanisms, epigenetic alterations contribute to a wide range of human diseases such as cancer, autoimmune disorders and NDDs (Moosavi and Motevalizadeh Ardekani, 2016). It has therefore been suggested that the epigenome serves as a “molecular bridge,” by which external triggers and stressors can modulate gene transcription and expression, and thereby contribute to an altered phenotype in the exposed individual (Nestler, 2009; Bollati and Baccarelli, 2010; Acevedo et al., 2021; Smeeth et al., 2021). The majority of studies that have researched epigenetic modifications within the MIA model focus on DNA methylation, which is consistently affected within the adult offspring brain (Labouesse et al., 2015; Richetto et al., 2017a,b; Schaafsma et al., 2017; Basil et al., 2018; Weber-Stadlbauer et al., 2021; Woods et al., 2021). However, despite the evidence that neural progenitors display dynamic changes in DNA methylation within the embryonic brain, the function of DNA methylation in neurogenesis has been questioned, where it is expected to play a lesser role in cell fate determination, and may, instead, act as a consequence of histone modifications (Stricker and Götz, 2018; Adam and Harwell, 2020).

Although there are a limited number of studies, there is some evidence that histone acetylation is dysregulated in the offspring brain following immune exposure (Tang et al., 2013; Pujol Lopez et al., 2016; Reisinger et al., 2016; Woods et al., 2021). Histone acetylation actively promotes neurogenesis through a number of histone acetyltransferases, such as the CREB binding protein and p300 co-activator family, which drive the differentiation of NECs by stimulating the transcription of pro-neural genes (Wang et al., 2010; Tsui et al., 2014; Yao and Jin, 2014; Schoof et al., 2019). Hence, a disturbance in this process, where the availability or regulation of these modifications is altered (as reported in the MIA offspring brain; Tang et al., 2013; Pujol Lopez et al., 2016; Reisinger et al., 2016), could provide a mechanism for the pro-neurogenic phenotype exhibited in MIA exposed animals. Nevertheless, as far as we know, no studies have investigated histone acetylation in the fetal MIA brain, which represents a major gap in the literature.

There are a number of hypothesized pathways in which an environmental stressor (such as MIA), may lead to altered epigenetic regulation in the exposed individual. First, regulation of epigenetic enzymes by cytokines has been reported, such as increased expression of DNA methyltransferase 1 by interleukin-6 (Braconi et al., 2010; Villagra et al., 2010), which is of interest following evidence of increased cytokine response in offspring brains acutely following insult (Woods et al., 2021). Second, availability of the substrate used by epigenetic enzymes to generate the modification may be limited. In the case of histone acetylation, acetyl-coenzyme A is utilized by histone acetyltransferases, which is produced following glycolysis in the mitochondria and is an essential intermediate of several metabolic pathways. In fact, mitochondrial biogenesis generates a number of substrates which alter epigenetic enzyme activity, including those responsible for DNA and histone modifications, as well as ATP-dependent chromatin remodelers (Wiese and Bannister, 2020). Recently, a growing body of literature has reported mitochondrial dysfunction in the MIA model (Robicsek et al., 2018; Swanepoel et al., 2018; Cieślik et al., 2020, 2021; Gyllenhammer et al., 2021; Zawadzka et al., 2021), which may provide a mechanistic pathway for altered epigenetic modifications and neurogenesis in the model.

Mitochondrial activity regulates the fate of neural progenitors, where a switch to oxidative phosphorylation from glycolysis, along with increased reactive oxygen species generation, promotes neuronal differentiation (Iwata and Vanderhaeghen, 2021). It is therefore possible that impaired mitochondrial activity, which is often concordant with elevated reactive oxygen species in the MIA model (Swanepoel et al., 2018; Cieślik et al., 2020, 2021), is controlling the acute switch from proliferative to neurogenic divisions in the embryonic brain. It is interesting to note that alternative studies of developmental insult, including mitochondrial dysfunction, intrauterine growth restriction and maternal hyperglycemia, report an almost identical phenotype in embryonic neurogenesis, where increased NECs are differentiating, at the expense of proliferation (Khacho et al., 2017; Ji et al., 2019; Brown et al., 2021). This suggests that disturbed neurogenesis may be the result of a common downstream pathway, such as oxidative stress, which has been linked to a wide range of diseases (Cenini et al., 2019; Forman and Zhang, 2021).

## 6. Conclusion

Neuronal development within the embryonic brain is clearly affected by MIA as demonstrated by an acute proliferation defect, which, in most cases, is concordant with increased differentiation of neurons and altered cortical lamination (Figure 2; Shi et al., 2009; De Miranda et al., 2010; Carpentier et al., 2011, 2013; Soumiya et al., 2011; Stolp et al., 2011; Chao et al., 2016; Gumusoglu et al., 2017; Wu et al., 2018; Ben-Reuven and Reiner, 2021; Canales et al., 2021).

Disturbed cortical neurogenesis has been linked to neural connectivity deficits in the postnatal offspring as well as behavioral phenotypes associated with ASD, such as reduced ultrasonic vocalizations (Wagner and MacDonald, 2021; Griffin et al., 2022), which has been reported in MIA offspring (Gzielo et al., 2021; Scott et al., 2021; Potter et al., 2023). Hence, the altered embryonic neurogenesis phenotype highlighted in this review may provide an underlying mechanism responsible for MIA-induced dysfunctional behavior. A recent review concluded that MIA causes defective hippocampal neurogenesis in the adult offspring, which is linked to defects in memory, mood and anxiety and is hypothesized to mediate susceptibility to future “hits” (Couch et al., 2021). Generation of the neurogenic niches in the adult brain, known as the SVZ and the subgranular zone, are dependent on the precise mechanisms of embryonic neurogenesis (Nicola et al., 2015; Mira and Morante, 2020). It could thus be postulated that the proliferation defect reported in this review, which remains defective at late gestational time points, is at the root of improper neurogenic niche development within the MIA model, as has been hypothesized following alternative developmental insults (Khacho et al., 2017; Brown et al., 2021). Accordingly, accelerated brain growth and dysregulated expression of genes involved in neurogenesis has been reported within ASD patients (Hazlett et al., 2011; Chow et al., 2012; Fan and Pang, 2017; Shen and Piven, 2017).

Studies of neurogenesis within the fetal MIA brain have mostly focused on the pallium (De Miranda et al., 2010; Gallagher et al., 2013; Braun et al., 2019; Canales et al., 2021), which is the main source of excitatory neurons within the cerebral cortex (Llorca and Deogracias, 2022). Yet, inhibitory interneurons, which are repeatedly reported to be affected following MIA and in NDDs (Nakamura et al., 2021; Yang et al., 2022; Yu et al., 2022), are predominantly derived from sub-pallial regions such as the caudal and medial ganglionic eminences as well as the preoptic area (Yang et al., 2022). To our knowledge, only one study has investigated neurogenesis within sub-pallial regions following MIA induction, which reported decreased expression of proliferation markers within the caudal ganglionic eminence during late gestation (Lacaille et al., 2019). Future studies will help to elucidate whether neurogenesis defects within sub-pallial regions are contributing to GABAergic deficits following MIA.

This review focused on the effect of MIA on embryonic neurogenesis and neuronal migration, which is critical for understanding the underlying mechanisms of MIA as well as establishing how MIA interacts with genetic or subsequent environmental insults to alter the neurodevelopmental trajectory. It is clear that the embryonic neurogenesis phenotype is significantly affected by the timing of MIA induction, consistent with the rapid nature of fetal brain development. Hence, further research is required to better understand how even seemingly minor alterations to the timing of immune insult affect neurogenesis and subsequent brain deficits. Sex-dependent variation should also be explored in order to understand how MIA differentially affects neurogenesis, which may be at the root of sex-specific brain and behavioral phenotypes. Future research in the field of neurogenesis will allow an improved mechanistic understanding of how MIA increases the risk of NDDs and hence will assist in therapeutic discovery.

## Author contributions

FM collated the literature, prepared the manuscript, and created the figures and tables. JG and RH supervised the manuscript preparation and suggested edits for the final draft. All authors contributed to the article and approved the submitted version.
